# Increasing Interest of Mass Communication Media and the General Public in the Distribution of Tweets About Mental Disorders: Observational Study

**DOI:** 10.2196/jmir.9582

**Published:** 2018-05-28

**Authors:** Miguel Angel Alvarez-Mon, Angel Asunsolo del Barco, Guillermo Lahera, Javier Quintero, Francisco Ferre, Victor Pereira-Sanchez, Felipe Ortuño, Melchor Alvarez-Mon

**Affiliations:** ^1^ Department of Psychiatry Clinica Universidad de Navarra University of Navarra Pamplona Spain; ^2^ Department of Surgery, Medical and Social Sciences University of Alcala Madrid Spain; ^3^ Instituto Ramón y Cajal de Investigaciones Sanitarias Madrid Spain; ^4^ Department of Medicine and Medical Specialities Hospital Universitario Príncipe de Asturias University of Alcala Madrid Spain; ^5^ Center for Biomedical Research in the Mental Health Network Madrid Spain; ^6^ Department of Psychiatry Hospital Universitario Infanta Leonor Complutense University Madrid Spain; ^7^ Department of Psychiatry Hospital Universitario Gregorio Marañón Complutense University Madrid Spain

**Keywords:** Twitter, social media, psychiatry, mental health

## Abstract

**Background:**

The contents of traditional communication media and new internet social media reflect the interests of society. However, certain barriers and a lack of attention towards mental disorders have been previously observed.

**Objective:**

The objective of this study is to measure the relevance of influential American mainstream media outlets for the distribution of psychiatric information and the interest generated in these topics among their Twitter followers.

**Methods:**

We investigated tweets generated about mental health conditions and diseases among 15 mainstream general communication media outlets in the United States of America between January 2007 and December 2016. Our study strategy focused on identifying several psychiatric terms of primary interest. The number of retweets generated from the selected tweets was also investigated. As a control, we examined tweets generated about the main causes of death in the United States of America, the main chronic neurological degenerative diseases, and HIV.

**Results:**

In total, 13,119 tweets about mental health disorders sent by the American mainstream media outlets were analyzed. The results showed a heterogeneous distribution but preferential accumulation for a select number of conditions. Suicide and gender dysphoria accounted for half of the number of tweets sent. Variability in the number of tweets related to each control disease was also found (5998). The number of tweets sent regarding each different psychiatric or organic disease analyzed was significantly correlated with the number of retweets generated by followers (1,030,974 and 424,813 responses to mental health disorders and organic diseases, respectively). However, the probability of a tweet being retweeted differed significantly among the conditions and diseases analyzed. Furthermore, the retweeted to tweet ratio was significantly higher for psychiatric diseases than for the control diseases (odds ratio 1.11, CI 1.07-1.14; *P*<.001).

**Conclusions:**

American mainstream media outlets and the general public demonstrate a preferential interest for psychiatric diseases on Twitter. The heterogeneous weights given by the media outlets analyzed to the different mental health disorders and conditions are reflected in the responses of Twitter followers.

## Introduction

Mental health disorders occur frequently in the general population. In 2015, approximately 44 million Americans suffered from some type of mental illness, with depression and anxiety representing the most prevalent forms [1]. Mental health disorders lead to a poor quality of life and patient disability [2,3]. Furthermore, mortality is significantly higher among people with mental health disorders than it is among comparable populations, with a 10-year median of potential life lost [4,5]. Additionally, mental health diseases commonly provoke self-stigma, societal stigma, or both, which negatively affect patients’ disclosure of these psychiatric disorders [6,7]. Social regard for mental health disorders appears to be a key factor for the adequate consideration of these diseases, for the understanding and support received by psychiatric patients, and for the funding provided for medical investment and research of these disorders [8]. Thus, measurement of the social relevance of mental disorders is a fundamental objective for progressing the field of psychiatry [9].

Access to, and the diffusion of news information, has dramatically changed in recent years. In addition to traditional media, the internet and social media have become pivotal instruments for sharing knowledge [10-12]. Accordingly, the internet has radically modified how most people find, communicate, and share information regarding health and medical conditions, and its use and popularity have increased considerably [13]. Its relevance is further exemplified by the growing reliance on the internet as a source of information and health advice [14]. Social media is a relatively new health communication channel that enables interactions among large groups of people suffering from the same afflictions and promotes the ability to find and share information about health and medical conditions and receive health messages [15].

For example, Twitter is a social networking site that is one of the most popular and widely used forms of social media [16,17] in which users (“tweeters”) post status updates (ie, “tweets”) that are distributed to “followers.” These tweets are also made available to the public. This form of largely public conversation in which “short bursts” of inconsequential information are relayed in 140-character “tweets” seems an unlikely source for lifelong learning [18,19].

Mainstream media outlets, such as television, radio, newspapers, and online journals are considered to be sensors and drivers of society [20-22]. These media outlets use Twitter as a tool for news distribution and thus subsequently inﬂuence large groups of people in real time [23]. The analysis of distributed tweets could represent an effective indicator of “real-world performance” [24-26]. Furthermore, because Twitter has become more popular, different players in health and medicine have begun to realize its potential for acquiring and distributing medical information [27]. Moreover, the qualitative and quantitative relevance of tweets has been shown in various investigations, including analyses of the interests and feelings of the general population with respect to health and disease, the interactions between patients and doctors or health care providers, and the promotion of the scientific impact of medical research in the news media. However, the validity of Twitter as a reflection of public opinion has been challenged [28-32]. Furthermore, patient attitudes toward various medical topics, including vaccines, illnesses, pain, drug use, and oncological and cardiovascular disease have been analyzed [33-41]. Consequently, the analysis of distributed tweets about mental health disease by primary media channels and the frequency of retweets they generate may be an effective tool for assessing social and individual interest toward psychiatric diseases.

In this paper, we investigated the distribution of tweets about mental health diseases from highly recognized and relevant American communication media sources. More specifically, the study cites periodicals and various television and radio channels, which are used as sensors of societal attitudes towards psychiatric disease throughout the first decade of Twitter’s networking activity. Furthermore, we analyzed the interest generated among followers through the quantification of subsequent retweets. As a control, we simultaneously studied the number of tweets distributed by our selected social media platforms about diseases considered to be the main causes of death within the United States of America (USA) as well as tweets about HIV because of its recognized social relevance.

## Methods

### Communication Media Analyzed

In this study, we focused on tweets sent among a representative sample of primary American communications media outlets. We selected 15 general media outlets among those with the highest number of followers on Twitter, as estimated by their individual accounts, and ranked among those with highest social influence during the study duration [42-46]. Furthermore, we selected representative samples from different categories of media outlets to avoid potential bias. We included 6 newspapers (*New York Times*, *Washington Post*, *Los Angeles Times*, *USA Today*, *Chicago Tribune*, and *New York Post*), 5 TV or radio channels (*NBC*, *CBS*, *Fox*, *CNN*, and *ABC*), 1 general magazine (*Time*), 1 news agency (*AP*), and 2 online news outlets (*BuzzFeed* and *Huffington Post*).

### Search Strategy

Our research strategy focused on searching for tweets that referred to common psychiatric terms of interest. We investigated all tweets sent from Twitter accounts, ﬁltering them according to speciﬁc criteria using the following list of keywords: anxiety, phobias, posttraumatic stress disorder (PTSD), panic disorder, generalized anxiety disorder (GAD), obsessive compulsive disorder (OCD), depressive disorder, suicide, bipolar disorder, insomnia, schizophrenia, attention deficit hyperactivity disorder or hyperactivity (ADHD), alcoholism, drug addiction, gambling disorder, anorexia nervosa, bulimia, dysthymia, addictions, addictive, Asperger syndrome, autism, personality disorder, and gender dysphoria. Additionally, as controls, we used tweets focused on the main causes of death in the USA (prostate, lung, colorectal, and breast cancer, stroke, diabetes mellitus, and chronic obstructive pulmonary disease [COPD]), the main causes of chronic neurologic degenerative disease (Alzheimer and Parkinson diseases) [47,48], and HIV infection.

### Search Tool Utilized

In this study, we used the Twitter Firehose data stream, which is managed by Gnip and allows access to 100% of all public tweets that match a set of “search” criteria (query) [49]. In our study, the search criteria were the previously indicated keywords, and the following is an example of a query: “depression -economic -great -tropical from:nytimes OR from:washingtonpost OR from:nypost OR from:latimes OR from:USATODAY OR from:chicagotribune OR from:CNN OR from:ABC OR from:NBCNews OR from:CBSNews OR from:FoxNews OR from:AP OR from:TIME OR from:HuffingtonPost OR from:BuzzFeed until:2017-01-01. Tweet Binder, the search engine we employed, uses automatic machine learning text analysis algorithms, and it also uses node.js and the PHP language, which enables an analysis of tweets in the json format (used by Gnip).

Next, all the collected tweets were individually inspected by 3 members of the research team to identify tweets deemed irrelevant for the purpose of this study. Tweets that included keywords not related to psychiatric content were excluded, such as those referring to suicide attacks, economic depression, etc. The content of the tweets was then specifically analyzed by 3 separate blinded members of the research team, and those with at least 2 coincidences were excluded. This process led to the creation of a more concise database that we could easily reference. Moreover, the number of tweets generated was stratified by month and year beginning in January 2007 and terminating in December 2016. We also analyzed the number of retweets that each tweet generated, which yielded a total database of 19,117 tweets and 1,455,787 retweets.

### Statistical Analysis

A descriptive analysis of the number of tweets and retweets was performed for both the mental health and control conditions. The correlations among the observation time units (months) were evaluated using the Spearman rank test. To analyze the retweets generated by the disease-related tweets, odds ratios (ORs) were calculated for each of the studied diseases. The odds of the sum of all conditions (retweet to tweet ratio) was used as the baseline and confidence intervals were calculated using a Bonferroni-adjusted significance level (alpha) of .001. To evaluate the annual changes within and differences between the two groups, a multivariable generalized linear model (negative binomial regression) was performed for both tweets and retweets. Finally, seasonality was studied through the Seasonal Decomposition procedure of a multiplicative time series model. All statistical analyses were performed using SPSS v22 and STATA v14.

## Results

### Media Outlets Showed a Marked Interest in Mental Health Diseases and Tweet Patterns Generated Responses From Followers

We first analyzed the number of tweets generated by 15 mainstream American media outlets related to mental health disorders beginning in 2007 (soon after the launch of Twitter) through December 2016. As a control, we also included a parallel analysis of tweets related to the primary causes of death in the USA (prostate, lung, colorectal, and breast cancer; stroke, diabetes mellitus, and COPD), the two most relevant chronic neurologic degenerative diseases (Alzheimer and Parkinson disease), and HIV infection.

As shown in Table 1, 13,119 tweets were generated by the media about mental health disorders. The number of tweets about each of the analyzed diseases follows a heterogeneous pattern of distribution, with a preferential accumulation for a select number of conditions. Suicide and gender dysphoria accounted for half of the total number of tweets. The tweets related to highly prevalent anxiety and its different clinical forms only accounted for 11.39% (1494/13119) of the total number, and it was followed by depression, which accounted for 10.66% (1399/13119) of tweets. Mental health diseases characterized by child and adolescent incidence, such as autism, Asperger syndrome, ADHD, anorexia, and bulimia, accounted for 13.87% (1819/13119) of the total tweets generated. Additionally, 9.39% (1232/13119) of all tweets were related to addictive disorders, specifically alcoholism, drug abuse, and gambling disorders. Less than 8% of the analyzed tweets referred to the eight other diseases included in the study. Of note, bipolar disorder and schizophrenia, both of which are highly prevalent and disabling, only accounted for 0.63% (82/13119) and 1.33% (174/13119) of all generated tweets, respectively.

In the parallel control study, we measured the tweets distributed by American media on the diseases that are considered to be the main causes of death in the USA and paradigmatic examples of diseases with a demonstrated level of social interest (Table 2). In total, only 5998 tweets were generated by social media on this group of prevalent and severe diseases. The number of tweets focused on each individual disease analyzed also followed a heterogeneous pattern of distribution. A predominance of tweets was observed for a select number of conditions. In total, 31.06% (1863/5998) of the tweets referred to the four most lethal forms of cancer, although they mainly focused on breast cancer (1321/5998, 22.02%). HIV infection and Alzheimer disease received 22.79% (1367/5998) and 17.56% (1053/5998) of the tweets about organic disease generated by social media, respectively. Additionally, 28.59% (1715/5998) of the tweets were related to diabetes mellitus, stroke, Parkinson disease, and COPD. However, despite its prevalence, COPD only accounted for 0.08% (5/5998) of the tweets.

**Table 1:** Number of tweets about mental health diseases distributed by American media and the retweets they generated. Percentages (%) were calculated with respect to the total number of tweets distributed about the mental diseases group and the retweets generated. Spearman rank correlation coefficients (rho) between the tweets and retweets are shown for each condition or disease along with the level of statistical significance.

Mental health condition or disease		Tweet, n (%)	Retweet, n (%)	Spearman rho	*P* value
Suicide		4124 (31.44)	268,395 (26.03)	0.876	<.001
Gender dysphoria		2555 (19.48)	238,298 (23.11)	0.941	<.001
**Total for anxiety disorders**		1494 (11.39)	134,726 (13.07)	0.907	<.001
	Anxiety	984 (7.50)	92,042 (8.93)	0.872	<.001
	PTSD^a^	453 (3.45)	39,243 (3.81)	0.991	<.001
	Phobias	34 (0.26)	1018 (0.10)	0.886	<.001
	GAD^b^	22 (0.17)	2386 (0.23)	0.172	.064
	Panic disorder	1 (0.01)	37 (<0.01)	–0.008	.927
Depression		1399 (10.66)	11,067 (11.26)	0.785	<.001
**Autism spectrum disorders**		1337 (10.19)	129,066 (12.52)	0.870	<.001
	Autism	1253 (9.55)	117,955 (11.44)	0.860	<.001
	Asperger syndrome	84 (0.64)	11,111 (1.08)	0.875	<.001
**Addictive disorders**		1232 (9.39)	83,809 (8.13)	0.822	<.001
	Addictions	933 (7.11)	67,114 (6.51)	0.798	<.001
	Alcoholism	146 (1.11)	7392 (0.72)	0.865	<.001
	Drug addiction	143 (1.09)	8997 (0.87)	0.865	<.001
	Gambling disorder	10 (0.08)	306 (0.03)	0.933	<.001
Anorexia and bulimia		274 (2.09)	11,792 (1.14)	0.852	<.001
ADHD^c^		208 (1.59)	12,103 (1.17)	0.853	<.001
Schizophrenia		174 (1.33)	15,232 (1.48)	0.839	<.001
Insomnia		128 (0.98)	10,014 (0.97)	0.825	<.001
Bipolar disorder		82 (0.63)	6946 (0.67)	0.867	<.001
OCD^d^		81 (0.62)	3564 (0.35)	0.907	<.001
Personality disorder		31 (0.24)	962 (0.09)	0.038	.684
Dysthymia		0 (0)	0 (0)	N/A^e^	N/A
Total for mental health disorders		13,119 (100)	1,030,974 (100)	0.915	<.001

^a^PTSD: posttraumatic stress disorder.

^b^GAD: generalized anxiety disorder.

^c^ADHD: attention deficit hyperactivity disorder.

^d^OCD: obsessive-compulsive disorder.

^e^N/A: not applicable.

Next, we investigated the impact of tweets about mental health and disease control among social media followers by analyzing the responses based on the number of retweets. In total, 1,030,974 retweets were related to the studied mental health diseases and 424,813 were related to the control organic diseases (Tables 1 and 2). We observed a significant correlation between the number of tweets referring to each individual mental health disorder and the number of subsequent retweets generated. The statistical significance of the correlations was similar for the control organic diseases. The percentages of tweets and retweets generated for each of the control diseases, mental health conditions, and psychiatric diseases are shown in a figure in Multimedia Appendix 1. A scatterplot of the tweets about mental health conditions, psychiatric diseases and control diseases as well as the number of retweets that they subsequently generated is also shown in the Multimedia Appendix 2.

**Figure 1:**
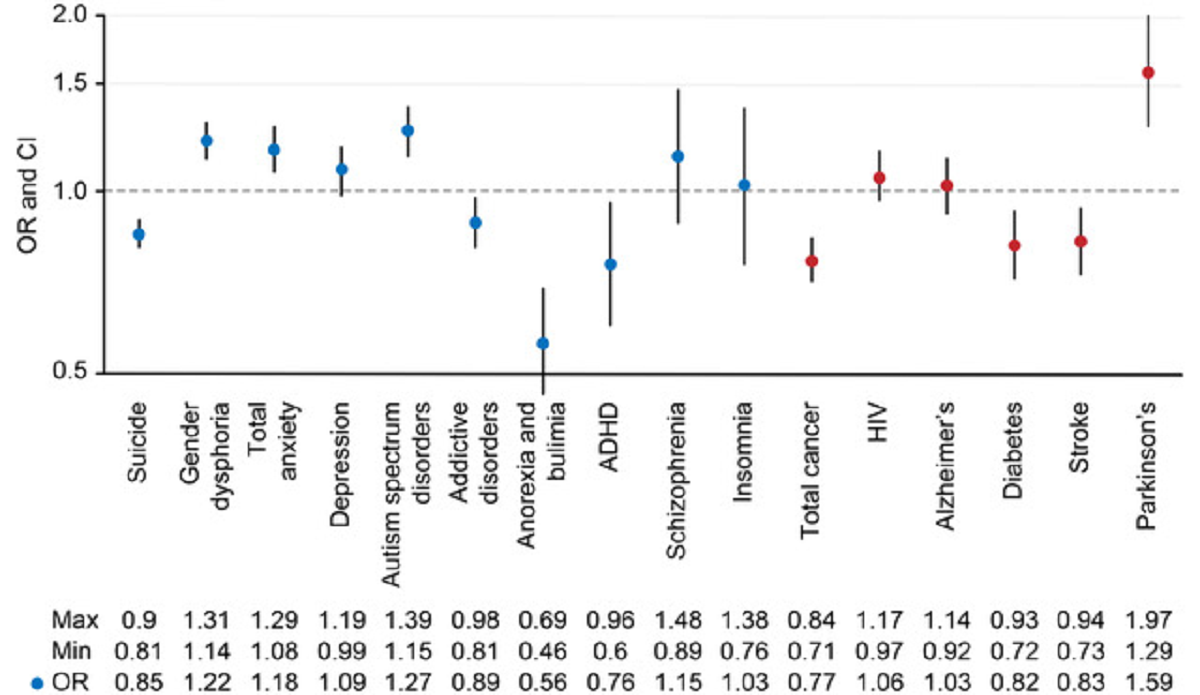
Different probabilities of retweets generated for tweets posted on mental health conditions and diseases (blue dots) and organic diseases (red dots). The odds ratios (ORs) are shown for the retweet to tweet ratios found for each individual disease with more than 100 tweets. Circles represent the calculated OR, and the vertical lines represent the CI. ADHD: attention deficit hyperactivity disorder.

We also investigated the retweets of disease-related tweets by analyzing the retweet to tweet ratio and absolute numbers for the mental health disorders and control diseases. We found that the retweet to tweet ratio for the psychiatric diseases was significantly higher than that found for the control diseases (OR 1.11, CI 1.07-1.14, *P*<.001). The analysis of the probabilities of retweeting a tweet related to a specific disease showed a marked heterogeneity between mental health and organic disorders (Figure 1). Among the mental health conditions and diseases, the tweets about suicide, addictive disorders, anorexia and bulimia, and ADHD had a statistically significantly lower probability of being retweeted. In contrast, the probability of being retweeted was significantly higher for tweets related to gender dysphoria, anxiety, and autism spectrum disorders. For the control diseases, we also found a heterogeneous pattern of retweet responses, with the highest statistically significant probability of being retweeted found for Parkinson disease. In contrast, the tweets about cancer, diabetes, and stroke had significantly lower probabilities of being retweeted.

**Table 2:** Number of tweets about control diseases distributed by American media and the retweets they generated. Percentages (%) were calculated with respect to the total number of tweets or retweets distributed in the control group of diseases. Spearman rank correlation coefficients between the tweets and retweets are shown for each condition or disease along with the level of statistical significance.

Control disease		Tweet, n (%)	Retweet, n (%)	Spearman rho	*P* value
**Total for cancers**		1863 (31.06)	109,697 (25.82)	0.715	<.001
	Breast cancer	1321 (22.02)	79,152 (18.63)	0.763	<.001
	Prostate cancer	326 (5.44)	13,675 (3.22)	0.648	<.001
	Lung cancer	196 (3.27)	16,425 (3.87)	0.733	<.001
	Colorectal cancer	20 (0.33)	445 (0.10)	0.845	<.001
HIV		1367 (22.79)	110,919 (26.11)	0.812	<.001
Alzheimer disease		1053 (17.56)	82,334 (19.38)	0.828	<.001
Diabetes		760 (12.67)	47,354 (11.15)	0.734	<.001
Stroke		701 (11.69)	44,328 (10.43)	0.796	<.001
Parkinson disease		249 (4.15)	30,160 (7.10)	0.873	<.001
COPD^a^		5 (0.08)	21 (<0.01)	0.624	<.001
Total for control diseases		5998 (100)	424,813 (100)	0.869	<.001

^a^COPD: chronic obstructive pulmonary disease.

**Figure 2:**
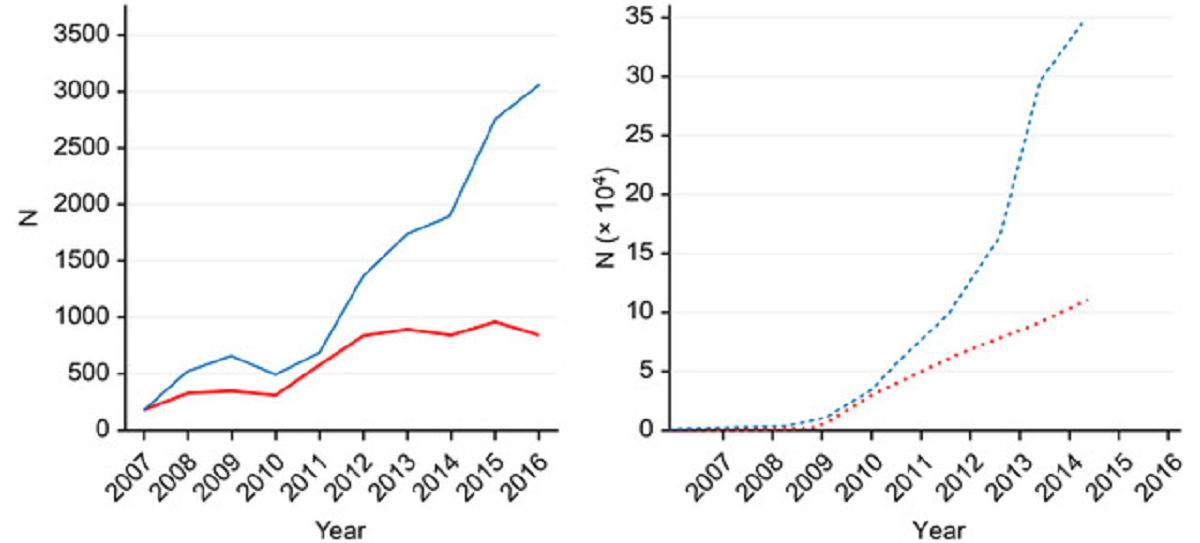
Kinetic study of the number of tweets (left panel, continuous line) distributed by American mass media outlets on mental health conditions and diseases (blue) and control diseases (red) and retweets (right panel, dotted line) generated by their followers; Y-axis: total number of tweets or retweets.

**Figure 3:**
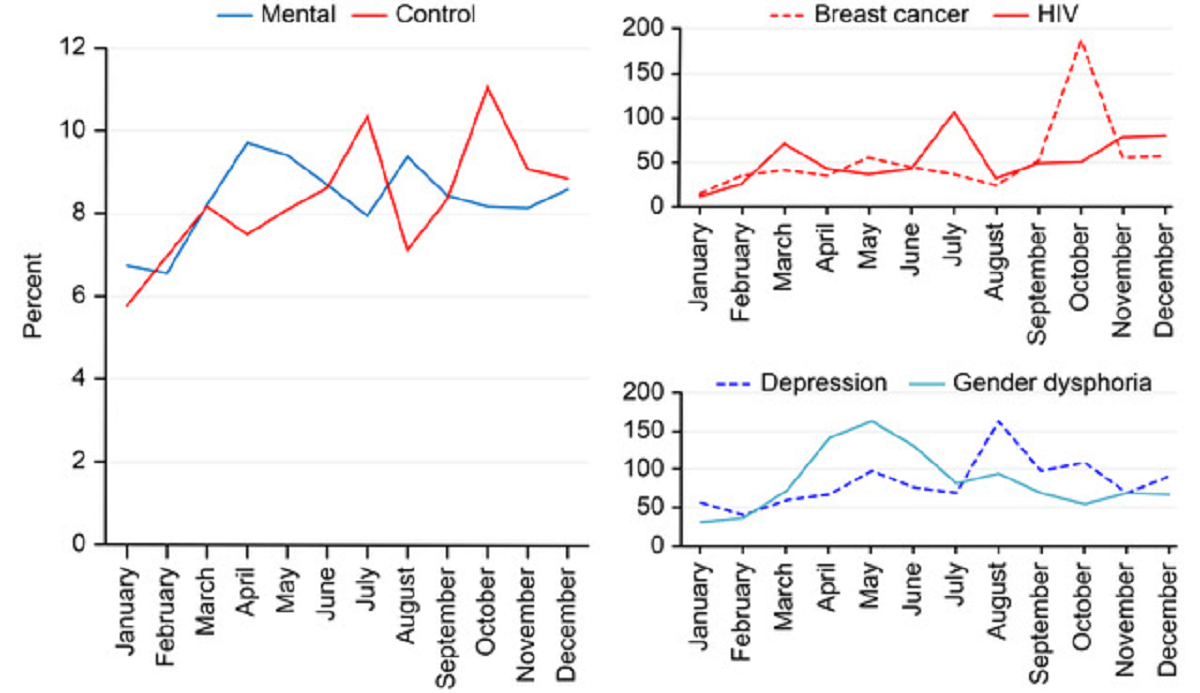
Left panel: monthly distribution of the tweets sent by American mass media outlets about mental health conditions and diseases (blue solid line) and control diseases (red solid line); Y-axis: percentages were calculated with respect to the total number of tweets. Top right panel: specific analyses of breast cancer and HIV infection; Y-axis: number of tweets. Bottom right panel: specific analyses of depression and gender dysphoria; Y-axis: number of tweets or retweets.

### Number of Mass Media Tweets and Follower Retweets Is Continuously Increasing

We analyzed the evolution of the number of tweets about mental health conditions and control diseases that were distributed by the mainstream American media outlets along the analyzed decade. We also studied the kinetics of the retweets that these tweets generated (Figure 2); and observed a steady and progressive increase in the number of tweets generated for mental health conditions and diseases by communication media across the analyzed years. Furthermore, there was an associated increase in the number of retweets sent by followers. Interestingly, a large increase in retweet responses was observed beginning in 2014. For the control diseases, an increase in the number of communication media generated tweets was observed between 2007 and 2012, and a steady level was reached by 2016. However, the number of generated retweets among nonpsychiatric control diseases also showed a continuous increase. To determine the effects of the year and type of disease, we ran generalized linear models for both tweets and retweets. In both models, these variables were statistically significant (*P*<.001). The output of the negative binomial regression parameters is included in the Multimedia Appendix 3.

We also investigated the number of tweets generated over continuous months about the mental health and control diseases. Temporal variability was observed in the frequency of tweets about psychiatric disease, with a significant increase in April and August and a decrease in February (Figure 3). Monthly variability in the tweets generated about organic control diseases was also observed, with a statistically significant increase in months July and October and a decrease in January. This monthly variability was also observed in the analysis of individual mental health conditions and diseases. The results obtained for gender dysphoria, depression, breast cancer, and HIV are shown as representative cases for both the mental health conditions and control diseases.

## Discussion

### Principal Findings

In this paper, we showed that American outlets show preferential interest in psychiatric disorders compared with prevalent and severe organic diseases. The elevated number of tweets sent by the analyzed media outlets about mental health conditions and diseases was heterogeneously distributed between the different clinical entities studied. The relative attention of media outlets for the different mental health disorders conditioned the retweet response of followers.

The important role of communication media outlets in generating popular opinion and emotions via information distribution has been clearly established in our society [50]. In addition to traditional forms of communication media, both the internet and social media have become particularly pivotal instruments for sharing knowledge and news. Along with this change in the pattern of access to and sharing of information, communicative mass media includes the use of social media for connecting to the public. Currently, the use of social media websites, such as Facebook and Twitter, is commonplace, with approximately 65% of American adults and 66% of British adults reporting ownership of at least one active social media account [51]. Likewise, Twitter is currently considered an equally effective channel for communication [52].

### Communication Media and Psychiatry

Our work demonstrates that American classic communication media outlets show a relevant interest in psychiatric diseases, as measured by the number of tweets about mental health conditions and disorders with respect to those about a group of severe and prevalent nonpsychiatric diseases, including the main causes of death in the USA. In recent decades, the stigma associated with mental health-related disorders has been widespread as evidenced by our social behaviors [53,54]. This social attitude has had major adverse effects on the lives of people with mental health problems, conditions, and diseases [55]. Therefore, the interest of traditional communication media outlets in psychiatric diseases should decrease over time. However, our findings contradict this hypothesis. The number of tweets sent about the analyzed mental conditions and diseases was higher than that of the control group throughout the decade examined, and a continuously increasing trend was observed in recent years. Interestingly, the control diseases included the main causes of mortality in the USA, such as the most predominant malignant tumor causes of death (cancer), stroke, diabetes mellitus, chronic degenerative neurological diseases, and COPD [47,48]. The control group of diseases also included HIV infection, a disease that has maintained a high level of general interest in our society in recent decades [56,57]. In addition to the demonstrated interest in mental health conditions and diseases by mass media, we found that this interest is more focused on certain clinical entities.

Interestingly, the relative weight given to each disease as defined by the percentage of tweets received was not related to the actual prevalence of the disease (the prevalence of mental health conditions, psychiatric diseases, and control diseases are included in the Multimedia Appendix 4). Despite the low incidence of suicide and gender dysphoria, these topics accounted for half of the tweets generated by communication media. In contrast, anxiety and depression are highly prevalent in society but only accounted for a quarter of the total number of tweets. Furthermore, psychiatric diseases with a marked prevalence and associated morbidity, such as schizophrenia and bipolar disorder, only accounted for a marginal percentage of the tweets. This lack of correlation between the prevalence and the morbidity or mortality (or both) of a disease and its relative presence in the number tweets generated by communication media outlets was also observed in the control group. These results are aligned with previous results demonstrating that certain chronic diseases, such as hypertension, are “undertweeted” relative to their prevalence, whereas other chronic diseases, such as diabetes and heart failure, are “over-tweeted” relative to their prevalence [58].

### Interest in Psychiatry on Twitter

The interest provoked by mental health disease-associated tweets sent by mass media organizations to the general public, as measured by the number of retweets generated by followers, is clearly relevant. The retweet frequency is a parameter that indicates the user interest in the topic of each tweet [59,60]. Our data demonstrate that the retweet to tweet ratio generated by mental health disease-related tweets was significantly higher than that of the control diseases. Thus, in addition to a correlation between the number of tweets sent about a specific disease and the retweet response provoked, the characteristics of the health disorder also modulate the interest and quantitative retweet response of the followers. This finding is clearly supported by the significantly higher possibility of retweeting a tweet on gender dysphoria, anxiety, and autism spectrum disorders and the decreased possibility of retweeting a tweet related to suicide, addictive disorders, anorexia and bulimia, and ADHD. Several reasons that are not mutually exclusive may explain this public behavior. First, the potential anonymity of Twitter might favor its use by people who present feelings of potential self-stigma. For example, tweeting about mental health conveys the notion of a “Twitter community” that allows communication to flourish, awareness to be raised, stigmas to be fought, and support to be both offered and received [51]. Twitter use allows for anonymity; thus, it is preferred by people with real or perceived personal and/or or social restrictions [61]. The reported use of Twitter by transgender individuals and allies to discuss health and social needs supports this statement [62]. Second, Twitter is becoming more popular in our society, and the average user profile is distributed across different age groups. However, Twitter is predominantly used by younger and middle-aged demographics [63,64]. Thus, the social media pattern of Twitter might indicate a modification in attitudes toward mental health diseases among these two generations. Furthermore, the age of the person affects their general interest in health-related matters [65]. Third, high rates of social media use are observed among individuals who experience mental health problems [66,67]. Fourth, health care professionals and provider communities may also show a greater interest in mental diseases and contribute to the dissemination of this information. However, the attitudes of professionals, such as general practitioners, towards these diseases cannot be considered optimal at the present time [68]. Additionally, certain mental health conditions, such as gender dysphoria and suicide, are topics that often appear in breaking social news and may easily go viral on Twitter. The information transmitted by mass media may be selected using different criteria, including content generally considered to be of public interest [69]. According to cultural selection theory, any selection of messages from communication media outlets will have a profound effect on society at large and can contribute to the modulation of individual and societal attitudes and knowledge [70]. Based on the frequency of tweets generated about mental health disorders found in this work, we conclude that mass media outlets do not support a quantitative stigmatic exclusion of psychiatric patients. However, the results observed for suicide should be further discussed. Suicide was one of the most frequently mentioned topics on Twitter by communication media outlets. Interestingly, the Werther effect of suicide reports in social media networks, such as Twitter, has been established [71]. Thus, the criteria applied for generating this increased frequency of suicide-related tweets by communication media outlets may require revision. Fortunately, a suicide-related tweet has a significantly reduced possibility of being retweeted by followers.

### Limitations

This study has some limitations. The relevance of Twitter as a marker of social interest is a matter of controversy [24-26,28-32]. Furthermore, news media outlets do not necessarily reflect the interests of society [72]. Large media outlets can also have a different set of priorities than news media in general. The newsworthiness of health science articles has previously been reported [73-75].

### Conclusions

In conclusion, our findings show a marked correlation between the number of tweets generated about a psychiatric or control disease and the number of retweets that are subsequently generated. These results could represent a coincidence between the interest of communication media outlets and the general population and/or merely the quantitative reactive response of followers to the tweets they receive. Interestingly, the frequency of retweeting a tweet related to suicide was less than expected, whereas that of gender dysphoria was greater. Moreover, there are contradictory results with respect to the association between mental health problems and social media, which indicates either the potential for harm or a significant improvement in social media engagement as previously described [71,76-78].

## Appendix

Multimedia Appendix 1 The percentages of tweets and retweets generated for each of the control diseases, mental health conditions, and psychiatric diseases.

Multimedia Appendix 2 A scatterplot of the tweets about mental health conditions, psychiatric diseases and control diseases as well as the number of retweets that they subsequently generated.

Multimedia Appendix 3 Output table for the negative binomial regression parameters.Estimated coefficients from IRR reports transformed into incidence-rate ratios.The standard errors (SEs) reported in the table were calculated using the robust or sandwich estimator of variance.

Multimedia Appendix 4 Prevalence of mental health conditions, psychiatric diseases, and control diseases.

## Abbreviations

ADHD: attention deficit hyperactivity disorder
COPD: chronic obstructive pulmonary disease
GAD: generalized anxiety disorder
OCD: obsessive-compulsive disorder
OR: odds ratio
PTSD: posttraumatic stress disorder
USA: United States of America
