# Supplementary figures and images for: Increasing Interest of Mass Communication Media and the General Public in the Distribution of Tweets About Mental Disorders: Observational Study
Source: J Med Internet Res. 2018 May 28;20(5):e205. doi: 10.2196/jmir.9582 (PMC5996178; doi:10.2196/jmir.9582)

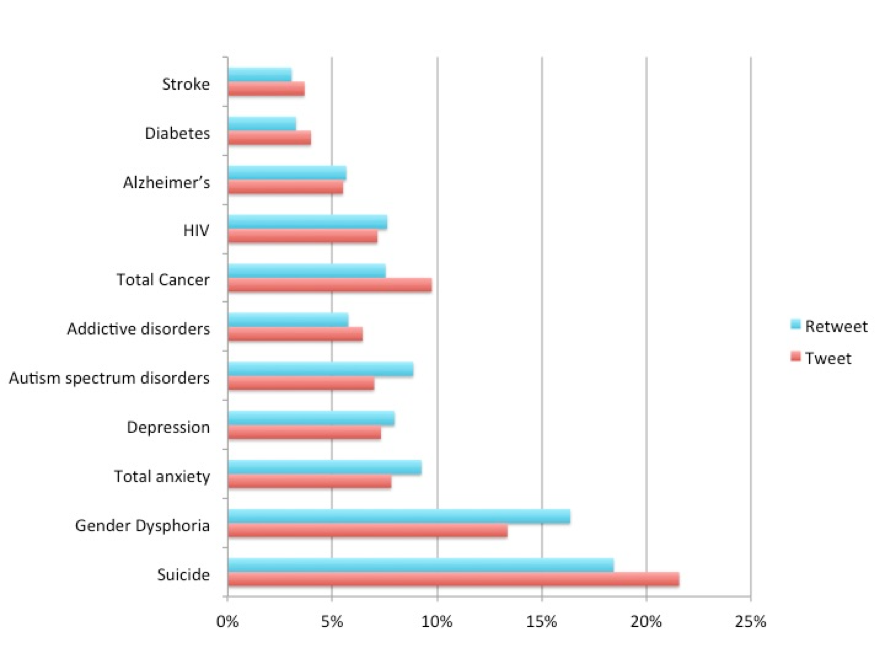

Supplement: Multimedia Appendix 1 [file jmir_v20i5e205_app1.png]

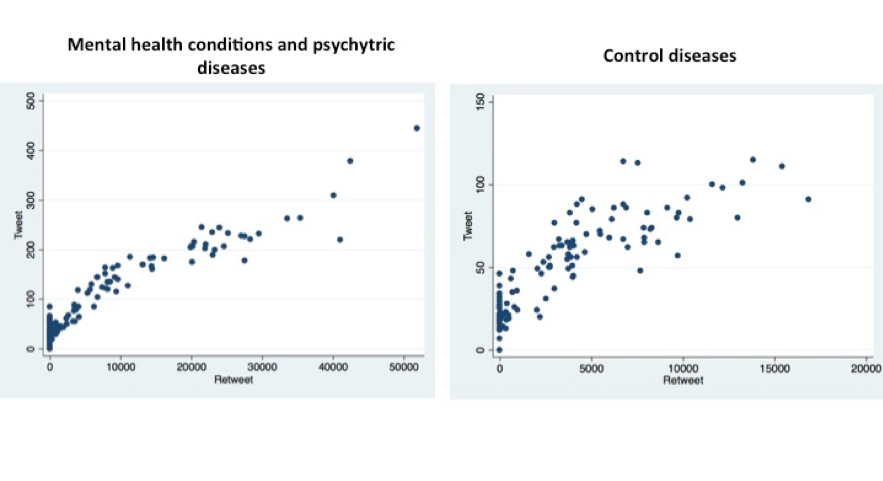

Supplement: Multimedia Appendix 2 [file jmir_v20i5e205_app2.png]

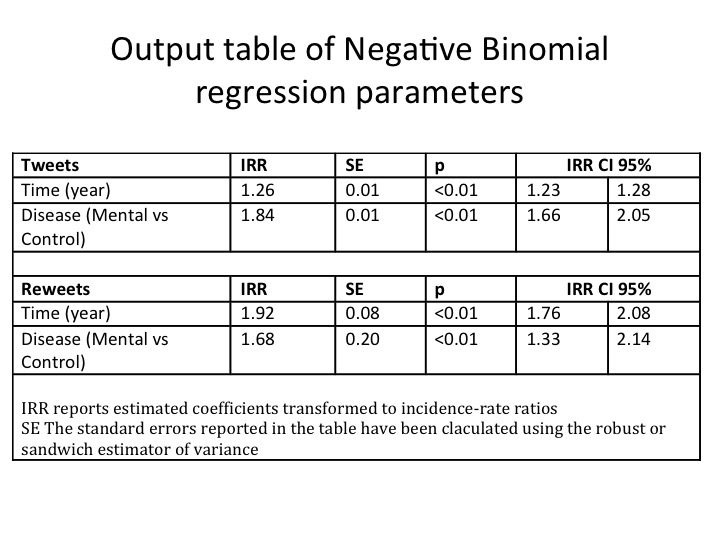

Supplement: Multimedia Appendix 3 [file jmir_v20i5e205_app3.png]

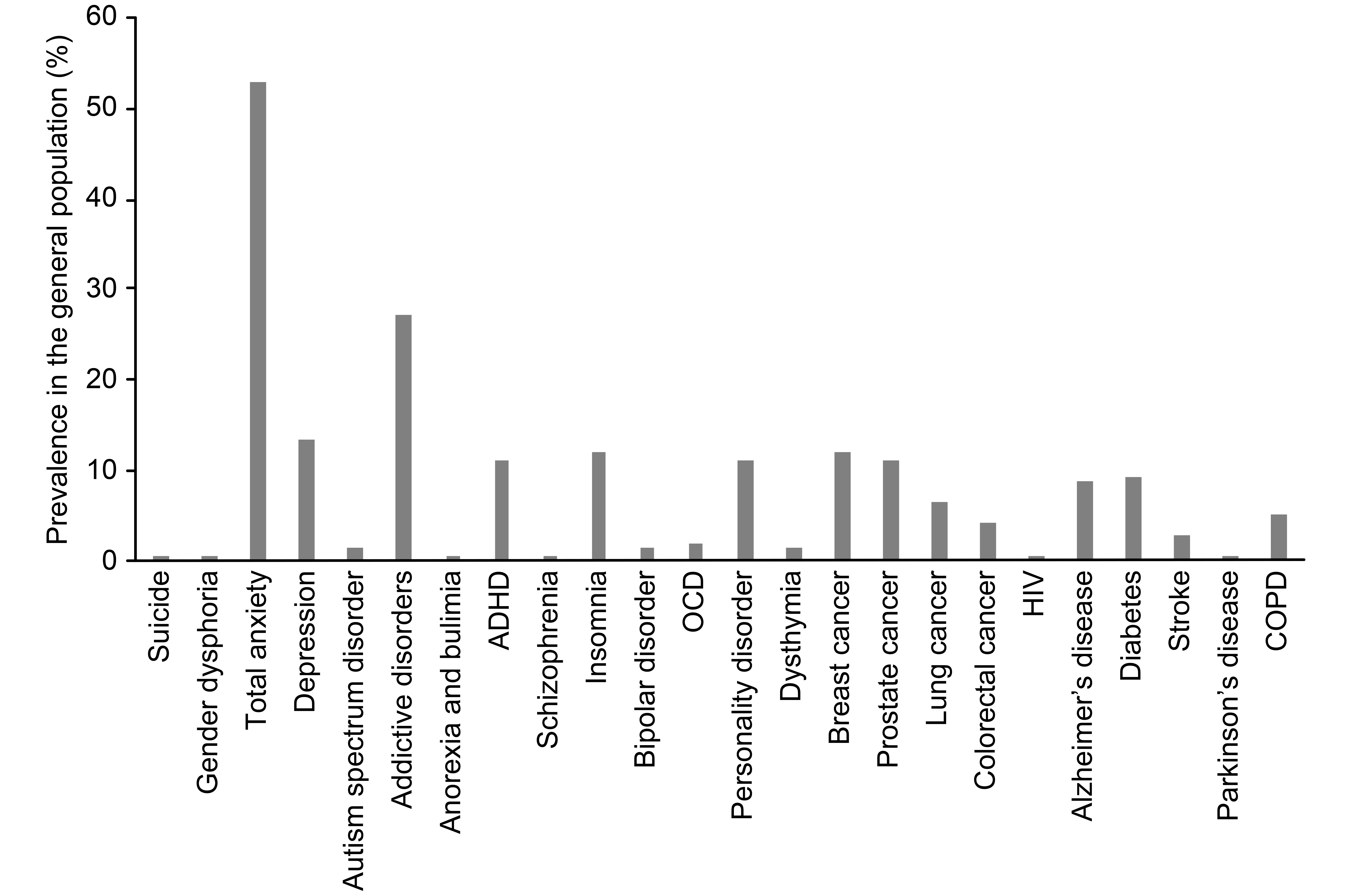

Supplement: Multimedia Appendix 4 [file jmir_v20i5e205_app4.png]
